# Skeletal health status among patients with chronic hypoparathyroidism: results from the Canadian National Hypoparathyroidism Registry (CNHR)

**DOI:** 10.1007/s00198-025-07410-7

**Published:** 2025-02-16

**Authors:** Aliya A. Khan, Hajar AbuAlrob, Dalal S. Ali, Zayd Al Kassem, Abdulrahman Almoulia, Habiba Afifi, Manoela Braga, Alice Cheng, Jouma Malhem, Adam Millar, Emmett Morgante, Parwana Muhammad, Terri L. Paul, Ally Prebtani, Zubin Punthakee, Tayyab Khan, Sarah Khan, Muhammad Shrayyef, Stan Van Uum, James Edward Massey Young, Maria Luisa Brandi, Michel Ovize, Blandine Weiss

**Affiliations:** 1 Division of Endocrinology and Metabolism, McMaster University, Hamilton, ON Canada; 2 Department of Health Research Methodology, McMaster University, Hamilton, ON Canada; 3 Bone Research and Education Centre, Oakville, Canada; 4 Department of Medicine, University of Toronto, Toronto, ON Canada; 5 The Division of Endocrinology and Metabolism, University of Toronto, Toronto, ON Canada; 6 Western University, London, ON Canada; 7 University of Toronto, Toronto, ON Canada; 8 Division of Otolaryngology–Head and Neck SurgeryDepartment of Surgery, McMaster University, Hamilton, ON Canada; 9 F.I.R.M.O. Onlus Italian Foundation for the Research On Bone Diseases, Florence, Italy; 10 Donatello Bone Clinic, Villa Donatello Hospital, Florence, Italy; 11 Amolyt Pharma, Ecully, France; 12 Divisions of Endocrinology and Metabolism, McMaster University, 3075 Suite #223 Hospital Gate, Oakville, ON L6M 1M1 Canada

**Keywords:** Fragility fracture, Hypoparathyroidism, Osteoporosis, Postmenopausal women, Skeletal health

## Abstract

**Summary:**

In the CNHR study, 35% of postmenopausal women had osteoporosis by BMD or fragility fracture, and 4% had both. Three men ≥ 50 had osteoporosis by BMD or fragility fracture (33.3%; *n* = 3/9). This suggests that close follow-up of skeletal health is necessary in postmenopausal women, and men ≥ 50 with chronic HypoPT.

**Purpose:**

Chronic hypoparathyroidism (HypoPT) has been associated with decreased bone turnover and abnormalities in bone mineral density (BMD), microarchitecture, and strength. Current guidelines do not recommend systematic evaluation of skeletal health in patients with chronic HypoPT. Our study assessed skeletal health in pre- and postmenopausal women with chronic HypoPT and adult men.

**Methods:**

This prospective study enrolled adults with chronic HypoPT from the Canadian National Hypoparathyroidism Registry. Clinical characteristics, bone fractures, biochemistry, and serum bone biomarkers were assessed at baseline. Skeletal health evaluation included assessments of fragility fractures, BMD at lumbar spine (LS), femoral neck (FN), total hip (TH), 1/3 radial sites, trabecular bone score (TBS), and bone biomarkers.

**Results:**

We present the baseline data of the patients enrolled in the registry. We analyzed a total of 101 patients: 18 men, 35 premenopausal, and 48 postmenopausal women. The mean (SD) age at the onset of HypoPT was 40.7 (16.8) years, and the average disease duration was 11.2 (8.6) years. The most common etiology was postsurgical (74.3% vs. 25.7% non-surgical). Most patients received calcium supplements (89%) and active vitamin D (80%) at baseline. No fragility fractures or low BMD were reported in premenopausal women. However, BMD at LS, FN, TH, and TBS were significantly lower in postmenopausal compared to premenopausal women.

**Conclusions:**

Overall, 35% of postmenopausal women had osteoporosis by BMD or prior fragility fracture, and 4% had both. Three men ≥ 50 years had osteoporosis by BMD or fragility fracture (33.3%; *n* = 3/9). This study suggests that close follow-up of skeletal health is necessary in postmenopausal women with chronic HypoPT and men ≥ 50 years.

**Supplementary Information:**

The online version contains supplementary material available at 10.1007/s00198-025-07410-7.

## Highlights

- Evaluate skeletal health in postmenopausal women with chronic HypoPT and men over the age of 50 years with BMD and X-rays of the thoracic and lumbar spine.
- Monitor skeletal health with BMD in postmenopausal women and men over the age of 50 years with chronic HypoPT.

**Supplementary Information:**

The online version contains supplementary material available at 10.1007/s00198-025-07410-7.

## Introduction

Chronic hypoparathyroidism (HypoPT) is a rare endocrine disorder characterized by hypocalcemia and hyperphosphatemia in the presence of low or inappropriately normal serum parathyroid hormone (PTH), which is the principal regulator of calcium and phosphate homeostasis [1–3]. The most common cause of chronic HypoPT is neck surgery (approximately 75% of cases), resulting in injury to the parathyroid glands or their blood supply [2, 4]. Non-surgical causes of chronic HypoPT (approximately 25% of cases) are frequently autoimmune, genetic, infiltrative, functional, post-radiation, or idiopathic [1, 2]. Chronic HypoPT has an estimated prevalence ranging from 6.4 to 37/100,000 person-years and an incidence reported to be 0.8 to 2.3/100,000/person-years [5].

The clinical presentation of chronic HypoPT is associated with the effects of hypocalcemia and hyperphosphatemia on multiple systems, including renal, neuromuscular, neurologic, behavioral/psychiatric, and cardiovascular systems [2]. Classic symptoms such as muscle cramps, paresthesia, brain fog, irritability, and depression are seen commonly. In severe cases, patients may experience arrhythmias, laryngospasm, bronchospasm, and seizures [2, 6, 7]. Long-term complications associated with chronic HypoPT include renal impairment, nephrocalcinosis, nephrolithiasis, cataracts, basal ganglia calcifications, cardiac arrhythmias, ischemic heart disease, and increased risk of infection [8–10]. Moreover, it has been recently reported that non-surgical HypoPT is associated with increased mortality and additional morbidities compared to the general population [11].

**Fig. 1:**
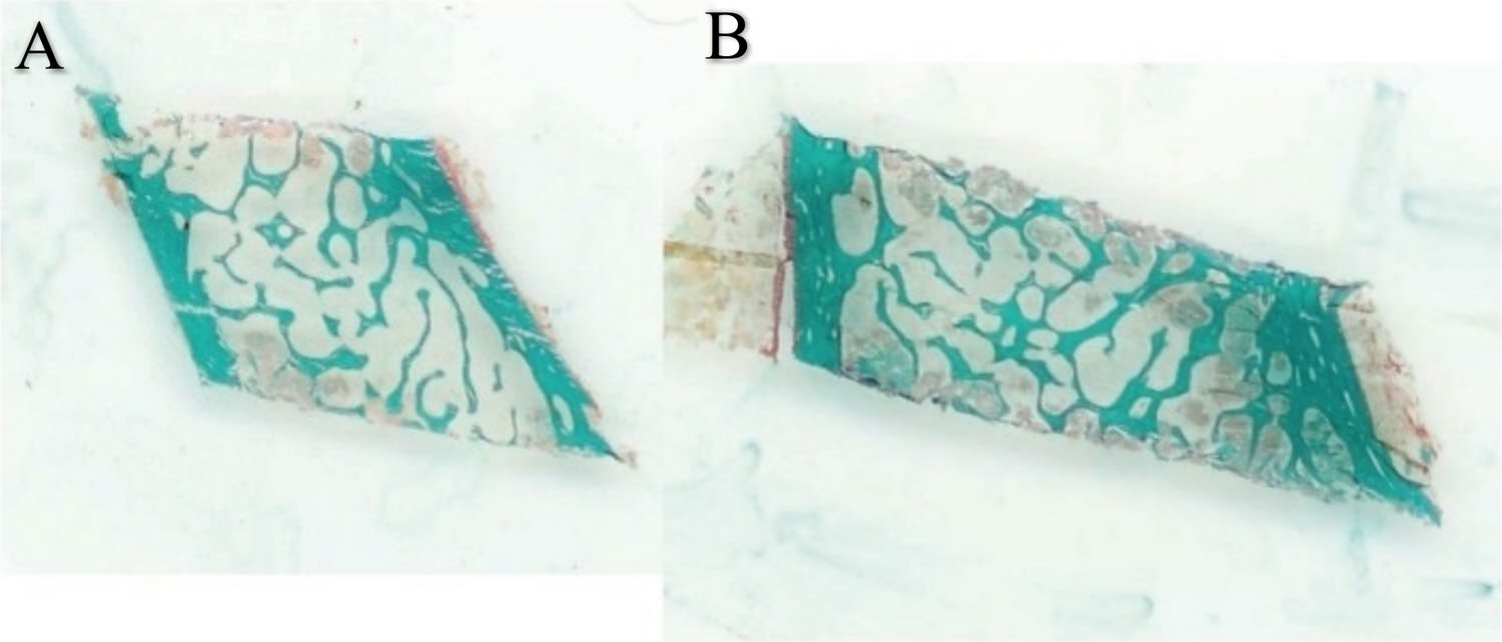
Low-power view of iliac crest bone biopsies from a control subject (**A**) and a subject with hypoparathyroidism (**B**) demonstrating the increase in cancellous bone volume and cortical thickness in the subject with hypoparathyroidism (Goldner trichome stain). Reproduced with permission from Rubin et al. [16]

**Fig. 2:**
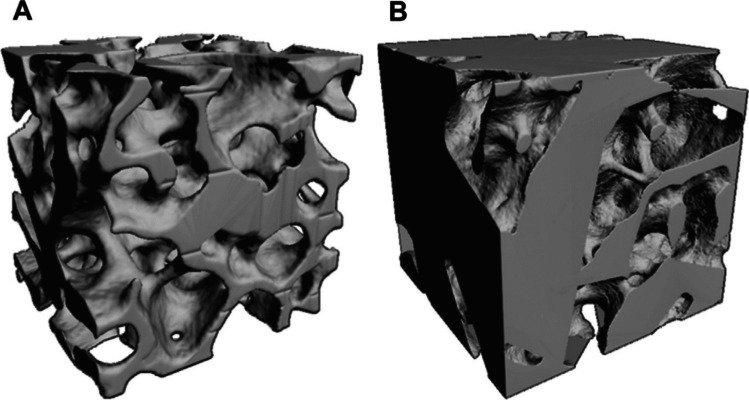
Reconstructed uCT images of cancellous bone from a control (**A**) and a subject with hypoparathyroidism (**B**). Note the dense trabecular structure in hypoparathyroidism. Reproduced with permission from Rubin et al. [20]

Chronic deficiency of PTH decreases bone turnover and is associated with abnormalities in bone density, microarchitecture, and possibly bone strength [7, 8, 12]. Several studies have shown a reduction in bone resorption and bone formation markers in patients with chronic HypoPT [8, 13, 14] whereas bone mineral density (BMD) values are usually higher in comparison to healthy eucalcemic subjects of the same age and sex [7, 8]. An increase in bone density in both cortical and cancellous compartments in patients with chronic HypoPT has been noted in dual-energy X-ray absorptiometry (DXA) studies and high-resolution peripheral quantitative computed tomography (HRpQCT) studies [15–19]; see Figs. 1 and 2 [16, 20]. However, when assessed by trabecular bone score (TBS), bone microarchitecture appears to be degraded in patients with chronic HypoPT regardless of BMD, as observed in those with postsurgical chronic HypoPT [21].

Histomorphometric data in patients with chronic HypoPT revealed reductions in bone mineralizing surface (58%), bone formation rate (80%), and remodelling activation frequency (54%) in comparison to eucalcemic age and sex-matched controls [22]. The resorption depth appears to be decreased, and the total resorption period is increased from 26 to 80 days, and in each remodelling cycle, more bone is formed than removed [22].

A systematic review and meta-analysis involving seven observational studies with a total of 1470 patients with chronic HypoPT showed that the risk of vertebral fractures is increased by twofold in patients with chronic HypoPT compared to healthy controls (OR 2.22, 95% CI 1.23, 4.03, *p* = 0.009, *I*^2^ = 49%, random-effects model) [23]. The observed increased risk of vertebral fractures was only noted in patients with non-surgical chronic HypoPT (OR 2.31, 95% CI 1.32, 4.03, *p* = 0.003, *I*^2^ = 3%, random-effects model) [23].

Current international guidelines do not specifically recommend regular follow-up of skeletal health in chronic HypoPT under conventional therapy [10]. Currently, there is limited information regarding skeletal health in individuals of all ages with chronic HypoPT with a potential underestimation of the effects of ageing and menopause on the skeleton.

New treatments of chronic HypoPT, such as PTH analogues, long-acting PTH, or PTHR1 agonists, have become recently available or are under development. Bone biomarkers and bone density data have recently been obtained and demonstrate the short-term impact of these treatments on bone turnover and bone density [24–26]. However, the long-term impact of drug therapy on skeletal health, especially in postmenopausal women at increased risk of bone fragility, remains unknown.

The aim of the present study was to systematically assess skeletal health in individuals with chronic HypoPT and, specifically, in postmenopausal women who represent a significant proportion of this population. The Canadian National Hypoparathyroidism Registry (CNHR) was established in 2014 with the objective of collecting epidemiologic data in Canadian patients with chronic HypoPT, including prevalence, management, treatment, and mortality [6]. The CNHR database was utilized to (1) evaluate the effects of chronic HypoPT on skeletal health in Canadian adults and (2) characterize the demographics, etiology, biochemical profile, symptom burden, and pharmacological management of this patient population.

## Methods

### Study design and population

This was a prospective, observational cohort study that included patients with chronic HypoPT from the CNHR. Patients of the CNHR were recruited from McMaster University, Western University, University of Toronto, and specialized academic as well as community centers across Canada. This study was approved by the McMaster University ethics review board, Hamilton Integrated Research Ethics Board, in 2014 [6].

We recruited adult men and women ≥ 18 years of age with chronic HypoPT, defined as low serum calcium in the presence of low PTH levels for at least 6 months prior to enrolment. Patients were receiving conventional therapy (i.e., calcium supplements and active vitamin D) for at least 6 months prior to enrolment or conventional therapy and PTH (1–34) or PTH (1–34) alone. Since PTH (1–84) is currently not available in Canada, patients treated with PTH (1–84) were not included. We excluded patients with transient HypoPT (i.e., resolved within 6 months of surgery), patients unable to provide informed consent, and patients currently participating in an interventional clinical study using an investigational drug. Patients with stable disease attended regular follow-up visits every 6–12 months or sooner if treatment adjustments were required.

### Baseline assessment

The following data were collected at baseline: demographics, age of disease onset, etiology of chronic HypoPT, clinical symptoms and comorbidities, history of bone fractures, and current and previous treatments for chronic HypoPT. Clinical laboratory tests included the following: serum PTH, total and ionized calcium, phosphorus, albumin, 25 hydroxyvitamin D (25(OH)D), 1,25 dihydroxyvitamin D (1,25(OH)_2_D), magnesium, thyroid stimulating hormone (TSH), creatinine, estimated glomerular filtration rate (eGFR; using the Chronic Kidney Disease Epidemiology Collaboration equation), calcium × phosphate product, 24-h urinary excretion of calcium and phosphorus, creatinine, and sodium. The serum bone biomarkers (procollagen 1 N-terminal propeptide [P1NP] and collagen type-I telopeptide [CTX]) were also evaluated.

### Areal bone mineral density, trabecular bone score, and fracture assessment

All patients prospectively underwent a DXA assessment evaluating BMD and TBS. The BMD study was completed by central analysis of DXA scans at four sites: lumbar spine (L1–L4), femoral neck, total hip, and 1/3 radial sites. Osteoporosis in men ≥ 50 years and postmenopausal women was defined as T-score ≤ − 2.5. Low BMD was defined as T-score < − 1 and > − 2.5 in men ≥ 50 years and postmenopausal women. Individuals with a Z-score < − 2 in men < 50 years and premenopausal women were described as having low BMD. Spine X-rays will be obtained prospectively every 2 years, as vertebral fracture assessment (VFA) is not consistently available at all collaborative centers.

### Statistical analysis

Categorical variables are summarized as the number and percentage of patients, and continuous variables are summarized using descriptive statistics as number of patients (*n*), mean, and standard deviation (SD). Correlation analysis was conducted by calculating the Pearson’s correlation coefficient where appropriate. All data were recorded and anonymized for analysis using Microsoft Excel®. Analysis was completed using SAS V9.4. A *p*-value of 0.05 was considered significant.

## Results

### Baseline characteristics

As of 29 February 2024, the study population consisted of 101 patients with chronic HypoPT: 18 (18%) men and 83 (82%) women, of whom 35 (42%) were premenopausal and 48 (58%) were postmenopausal women. This study included patients treated with both conventional therapy and PTH (1–34) (teriparatide). Our analysis specifically focused on those receiving conventional therapy. We also conducted a sensitivity analysis, which showed no significant differences between the group receiving conventional therapy and the group receiving PTH (1–34) therapy. Therefore, all patients remained in the final analysis. Baseline characteristics for the study population and by subgroups are presented in Table 1.

**Table 1:** Baseline characteristics of study participants by etiology (*N* = 101)

Etiology	*N*		*N*	Mean ± SD	Minimum	Maximum
Non-surgical	26	Age (yrs)	26	44.1 ± 21.1	22.00	85.00
		Age at menopause (yrs)	6	49.1 ± 7.6	35.00	56.00
		Age of disease onset (yrs)	24	27.6 ± 24.1	0.00	78.00
		Duration of disease (yrs)	24	13.8 ± 12.1	0.00	44.00
		Duration of postmenopausal status (yrs)	5	18.4 ± 12.2	2.00	34.00
		BMI (kg/m2)	23	25.2 ± 4.2	17.70	35.70
		Gender	***N***	**%**		
		*Females*	19	73.08		
		*Males*	7	26.92		
		Menopause status (female *N* = 19)				
		*Premenopausal*	11	57.89		
		*Postmenopausal*	8	42.11		
		Age				
		*Age 50 yrs or more*	9	34.62		
		*Age less than 50 yrs*	17	65.38		
Postsurgical	75	Age (yrs)	75	55.7 ± 12.5	33.00	82.00
		Age at menopause (yrs)	33	49.8 ± 5.1	38.00	59.00
		Age of disease onset (yrs)	74	45.0 ± 10.7	24.00	71.00
		Duration of disease (yrs)	73	10.3 ± 7.0	0.00	32.00
		Duration of postmenopausal status (yrs)	30	14.8 ± 9.7	1.00	42.00
		BMI (kg/m^2^)	64	30.1 ± 8.5	16.50	56.20
		Gender	***N***	**%**		
		*Females*	64	85.33		
		*Males*	11	14.67		
		Menopause status (female *N* = 64)				
		*Premenopausal*	24	37.50		
		*Postmenopausal*	40	62.50		
		Age				
		*Age 50 yrs or more*	50	66.67		
		*Age less than 50 yrs*	25	33.33		

Data are presented as mean ± SD or *n* (%)

As expected, the most common etiology was postsurgical, reported in 74.3% of patients (*n* = 75). Of these, 46 cases (61.3%) were associated with surgery for thyroid cancer, while 29 cases (38.7%) were related to surgery for benign thyroid or parathyroid disease. A non-surgical etiology was reported in 25.7% (*n* = 26) of patients, with 57.7% (*n* = 15) being idiopathic and 42.3% (*n* = 11) associated with genetic variants including *CaSR*, *GATA3*, *GCM2*, *PTHR*, and *TBX1* genes. An autoimmune etiology (*AIRE* gene variant) was identified in one patient (9.1%, *n* = 1/11).

The most commonly reported symptoms were paresthesia in 44.6% of patients (*n* = 45), numbness in 39.6% of patients (*n* = 40), and muscle cramps in 33.7% of patients (*n* = 34). Fatigue and muscle spasm were reported in 16.8% of patients (*n* = 17). There was no significant difference in serum calcium levels among participants with and without paresthesia or numbness (see supplementary Tables 1 and 2). Less frequent symptoms were confusion (10%, *n* = 10), seizure (9%, *n* = 9), depressed mood (3.0%, *n* = 3), and laryngospasm (1.0%, *n* = 1).

Regarding comorbidities, hypothyroidism was the most frequent (66%, *n* = 67) followed by hypertension (31.7%, *n* = 32). Chronic kidney disease and cardiovascular disease were reported in 12% (*n* = 12) and 9% (*n* = 9) patients, respectively. Less frequent comorbidities were osteoarthritis (4.0%, *n* = 4), chronic obstructive pulmonary disease (1.0%, *n* = 1), and hyperthyroidism (1.0%, *n* = 1).

The majority of patients with chronic HypoPT were receiving oral calcium supplements (89%; mean (SD) dose 2080 (1828) mg/day), calcitriol (80%; mean (SD) dose 0.7 (0.7) µg/day), LT4 (synthroid/levothyroxine) was administered in 66.3% of patients (*n* = 67) at a mean (SD) dose of 134.0 (89.0) µg/day, and oral magnesium supplement was administered to 24.8% of patients (*n* = 25) at a mean (SD) dose of 968.4 (1135.4) mg/day. Cholecalciferol supplementation was taken by 65.4% of patients at a mean (SD) dose of 2119.9 (1553.1) IU/day. In total, 11% of patients (*n* = 11) received PTH (1–34) (teriparatide) at a mean (SD) dose of 18.9 (8.0) µg/day. Only postmenopausal women were on antiresorptive therapy for concomitant osteoporosis. Seven were on bisphosphonate therapy (alendronate) (14.5%, 7/48), and 3 were on denosumab (6%, 3/48).

### Biochemical profile according to menopausal status

The biochemical profile of premenopausal and postmenopausal women with chronic HypoPT is shown in Table 2. Total and corrected calcium, alkaline phosphatase (ALP), and 25(OH)D were significantly higher in postmenopausal compared to premenopausal women, whereas eGFR and TSH were significantly lower in postmenopausal than in premenopausal women. We found no statistically significant difference in serum calcium levels, phosphorus, magnesium, and 25(OH)D levels among men and postmenopausal women (see supplementary Tables 3 and 4). The bone biomarkers CTX and P1NP were higher in postmenopausal women; however, this was not statistically significant in comparison to premenopausal women.

**Table 2:** Biochemical variables and bone markers among premenopausal and postmenopausal women

	Premenopausal women		Postmenopausal women		*p*-value
	Mean ± SD	Range	Mean ± SD	Range	
Total calcium (mmol/L)	2.11 ± 0.28 (*n* = 35)	1.15–2.38	2.22 ± 0.18 (*n* = 48)	1.67–2.59	**0.030**
Corrected calcium (mmol/L)	2.03 ± 0.26 (*n* = 35)	1.15–2.42	2.14 ± 0.17 (*n* = 48)	1.53–2.49	**0.023**
Ionized calcium (mmol/L)	1.14 ± 0.09 (*n* = 32)	0.95–1.27	1.15 ± 0.11 (*n* = 43)	0.80–1.39	0.437
Calcium phosphate product	2.74 ± 0.63 (*n* = 30)	1.40–4.09	2.93 ± 0.52 (*n* = 45)	1.58–4.30	0.149
Magnesium (mmol/L)	0.80 ± 0.08 (*n* = 32)	0.53–0.94	0.81 ± 0.08 (*n* = 48)	0.55–1.04	0.365
Phosphate (mmol/L)	1.35 ± 0.23 (*n* = 31)	0.77–1.82	1.36 ± 0.25 (*n* = 46)	0.74–2.00	0.876
eGFR (mL/min)	94.63 ± 18.35 (*n* = 35)	46.00–120.00	73.94 ± 23.24 (*n* = 48)	8.00–116.00	** < 0.0001**
ALP (U/L)	65.58 ± 28.45 (*n* = 31)	13.00–151.00	77.89 ± 22.85 (*n* = 45)	10.00–125.00	**0.040**
PTH (pmol/L)	1.71 ± 1.90 (*n* = 34)	0.30–11.20	1.77 ± 1.09 (*n* = 46)	0.30–4.30	0.845
TSH (mL/L)	3.78 ± 4.65 (*n* = 35)	0.01–18.80	1.88 ± 2.24 (*n* = 46)	0.02–9.38	**0.018**
25(OH)D (nmol/L)	90.08 ± 23.38 (*n* = 33)	44.00–164.00	106.29 ± 25.89 (*n* = 45)	54.00–162.00	**0.006**
1,25(OH)D (pmol/L)	98.05 ± 37.42 (*n* = 20)	36.00–171.00	104.26 ± 39.69 (*n* = 23)	23.00–216.00	0.602
CTX^a^ (ng/L)	242.41 ± 214.37 (*n* = 22)	65.00–1015.00	346.06 ± 495.31 (*n* = 36)	71.00–3051.00	0.358
P1NP^b^ (µg/L)	37.57 ± 40.10 (*n* = 23)	11.00–215.00	47.11 ± 33.64 (*n* = 34)	10.00–170.00	0.335
24 h Urine calcium (mmol/day)	6.00 ± 6.56 (*n* = 25)	0.26–30.40	4.84 ± 3.55 (*n* = 41)	0.53–14.61	0.356

*1,25(OH)D* 1,25-dihydroxyvitamin D, *25(OH)D* 25-hydroxyvitamin D, *ALP* alkaline phosphatase, *CTX* collagen type-I telopeptide, *eGFR* estimated glomerular filtration rate, *P1NP* procollagen 1 N-terminal propeptide, *PTH* parathyroid hormone, *TSH* thyroid stimulating hormone. Bold indicates statistically significant *p*-values

^a^Normal reference range of CTX in premenopausal women 136–689 ng/L, postmenopausal women 177–1015 ng/L

^b^Normal reference range of P1NP in premenopausal women 19–83 µg/L, postmenopausal women 16–98 µg/L

Hypercalciuria (> 7.5 mmol/day) was observed in 19 patients (18.8%), including 4 men, 6 premenopausal women, and 9 postmenopausal women. The incidence of nephrolithiasis was 16.7% in the study population (*n* = 10/60), mostly affecting postsurgical chronic HypoPT and postmenopausal women. The incidence of nephrocalcinosis was 8.3% (*n* = 5/60), and the majority of cases (*n* = 4/5) were postmenopausal women.

Further subgroup analyses by sex (women vs. men) and etiology (non-surgical vs. postsurgical chronic HypoPT) revealed a similar biochemical profile among subgroups, with few statistically significant differences between subgroups (Supplementary Tables 5 and 6). Creatinine was significantly lower among women in comparison to men, likely due to sex-related variations, as men typically have higher creatinine levels than women [27–29]. Men and women had comparable eGFR, whereas 1,25(OH)_2_D was significantly higher in women. Corrected serum calcium level was significantly higher in patients with postsurgical compared with non-surgical chronic HypoPT.

### Skeletal health status (BMD and TBS)

In this study population of 101 patients with chronic HypoPT, mean (SD) BMD values were as follows: 1.212 (0.225) gm/cm^2^ at L1–L4 (*n* = 85), 0.987 (0.192) gm/cm^2^ at femoral neck (*n* = 88), 1.054 (0.207) gm/cm^2^ at total hip (*n* = 87), and 0.840 (0.134) gm/cm^2^ at 1/3 radius (*n* = 80). The TBS was within the normal range, with a mean (SD) of 1.33 (0.15) (*n* = 69) (TBS classification: NR in postmenopausal women > 1.350, partially degraded (1.200 < TBS < 1.350), or degraded TBS ≤ 1.200) [30, 31].

A correlation analysis of BMD and TBS among premenopausal and postmenopausal women with chronic HypoPT is presented in Table 3. Total hip, L1–L4, and femoral neck BMD were significantly lower in postmenopausal than in premenopausal women, whereas BMD values at 1/3 radius were similar between subgroups. TBS was significantly lower in postmenopausal than premenopausal women (1.29 (0.16) vs. 1.41 (0.10); *p* = 0.001). For a comprehensive evaluation of skeletal health status in this patient population, Z-scores and T-scores were also assessed in premenopausal and postmenopausal women, respectively (Table 3).

**Table 3:** BMD and TBS by menopausal status

	Premenopausal women		Postmenopausal women		*p*-value
	Mean ± SD	Range	Mean ± SD	Range	
Total hip BMD (gm/cm^2^)	1.077 ± 0.69 (*n* = 31)	0.736–1.476	0.974 ± 0.202 (*n* = 40)	0.669–1.416	**0.025**
Total hip Z-score^a^	0.870 ± 1.286 (*n* = 30)	− 1.500–3.400	-	-	-
Total hip T-score^b^	-	-	− 0.153 ± 1.661 (*n* = 40)	− 2.600–3.700	-
L1-L4 BMD (gm/cm^2^)	1.268 ± 0.155 (*n* = 30)	0.992–1.538	1.136 ± 0.232 (*n* = 39)	0.738–1.753	**0.009**
L1-L4 Z-score^a^	0.983 ± 1.188 (*n* = 29)	− 1.000–3.100	-	-	-
L1-L4 T-score^b^	-	-	− 0.192 ± 1.905 (*n* = 39)	− 3.700–4.800	-
Femoral neck BMD (gm/cm^2^)	1.010 ± 0.170 (*n* = 32)	0.596–1.362	0.914 ± 0.187 (*n* = 40)	0.542–1.393	**0.028**
Femoral neck Z-score^a^	0.587 ± 1.172 (*n* = 31)	− 1.900–2.900	-	-	-
Femoral neck T-score^b^	-	-	− 0.420 ± 1.594 (*n* = 40)	− 2.900–4.700	-
1/3 radius BMD (gm/cm^2^)	0.842 ± 0.106 (*n* = 31)	0.582–0.990	0.791 ± 0.121 (*n* = 36)	0.483–0.958	0.077
1/3 radius Z-score^a^	− 0.037 ± 0.773 (*n* = 30)	− 1.900–1.200	-	-	-
1/3 radius T-score^b^	-	-	− 0.900 ± 1.233 (*n* = 36)	− 3.500–1.700	-
TBS score^c^	1.408 ± 0.102 (*n* = 26)	1.089–1.622	1.288 ± 0.155 (*n* = 32)	0.968–1.562	**0.001**

*BMD* bone mineral density, *L* lumbar spine, *TBS* trabecular bone score, *SD* standard deviation. Bold indicates statistically significant *p*-values

^a^Z-scores were calculated in premenopausal women

^b^T-scores were calculated in postmenopausal women

^c^Degraded TBS is ≤ 1.23, partially degraded is > 1.23 but ≤ 1.31, and normal TBS is > 1.31

Additionally, subgroup analyses of BMD and TBS by sex (women vs. men) and etiology (non-surgical vs. postsurgical chronic HypoPT) were performed (supplementary Tables 7 and 8). Statistically significant differences were observed between men and women with chronic HypoPT in BMD values at total hip, femoral neck, and 1/3 radius; these values were consistently lower in women than in men. The analysis of patients with non-surgical and postsurgical chronic HypoPT revealed comparable BMD values at all sites between subgroups, but a significantly lower TBS in postsurgical than in non-surgical patients.

### Low BMD, osteoporosis, and fragility fractures

Overall, 11.9% of patients (*n* = 12/101) reported a history of fragility fractures, most of whom were postmenopausal women (83.3%, *n* = 10/12). The other 2 patients were men ≥ 50 years (16.7%, *n* = 2/12). Location of fracture was reported in 12 patients, including hip (*n* = 4), vertebrae (*n* = 2), ribs (*n* = 2), wrist (*n* = 1), humerus (*n* = 1), and clavicle (*n* = 2). The prevalence of fragility fractures in postmenopausal women was 20.8% (*n* = 10/48), and it was 22.2% (*n* = 2/9) in men ≥ 50 years. No fragility fractures were reported in premenopausal women or men under age 50 years.

Low BMD was confirmed by T-score < − 1.0 and > − 2.5 at any site in postmenopausal women and men ≥ 50 years, and by Z-score ≤ − 2 at any site in premenopausal women and men < 50 years. Overall, 12 patients (11.9%, *n* = 101) showed low BMD, all of whom were postmenopausal women. The prevalence of low BMD in postmenopausal women was 25.0% (*n* = 12/48). Low BMD was not present in premenopausal women or men in this cohort.

**Fig. 3:**
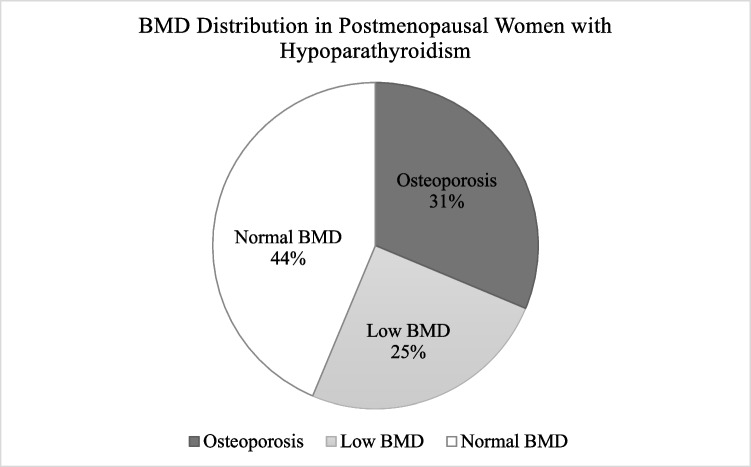
Distribution of postmenopausal women with low BMD or osteoporosis. Low BMD is defined as a T-score > −2.5 and lower < −1.0 or osteoporosis as defined by a T-score ≤ − 2.5 at any one of the following sites: lumbar spine, femoral neck, total hip or 1/3 radial site

In total, 15 postmenopausal women (31.3%, *n* = 48) and 2 men ≥ 50 years (22.2%, *n* = 9) showed osteoporotic T-scores (≤ − 2.5) at any site. The distribution of patients with low BMD and osteoporosis is presented in Fig. 3. The correlation between the duration of disease and BMD variables has been evaluated. The results indicate statistically significant positive correlations between the duration of disease and the absolute BMD in gm/cm^2^ at the following sites: L1–L4 BMD (*r* = 0.317, *p* = 0.0034), femoral neck BMD (*r* = 0.272, *p* = 0.0107), and total hip BMD (*r* = 0.280, *p* = 0.0090) (Table 4). There was no correlation at the 1/3 radial site.

**Table 4:** Correlation analysis between duration of disease and BMD

Variable	*N*	Correlation coefficient (*r*)	*p*-value
L1-L4 BMD (gm/cm^2^)	84	0.317	0.0034
Femoral neck BMD (gm/cm^2^)	87	0.272	0.0107
Total hip BMD (gm/cm^2^)	86	0.280	0.0090
1/3 radial BMD (gm/cm^2^)	79	0.091	0.4230

We further analyzed the proportion of patients with an osteoporotic T-score at any site and/or fragility fracture. In total, 19.8% of patients (*n* = 20/101; 17 postmenopausal women and 3 men ≥ 50 years) had an osteoporotic T-score or fragility fracture(s), whereas 4.0% patients (*n* = 4/101; all postmenopausal women) had both. Overall, 35% of postmenopausal women had osteoporosis by BMD or fragility fracture, and 4% had both. Three men over ≥ 50 years had osteoporosis by BMD or fragility fracture (33.3%, *n* = 3/9).

None of the men under age 50 years had osteoporosis or a prior fragility fracture.

### Subgroup analysis of PTH (1–34) users versus non-PTH users

The majority of the PTH (1–34) users were postmenopausal women (80%), with an average age of 64 ± 16.2 years. The primary etiology was postsurgical HypoPT (63.6%), and the average duration of chronic HypoPT was 17.1 ± 11.3 years. All patients were treated with PTH (1–34). Dosage regimens varied, with 36.4% receiving 20 mcg daily, and others receiving less frequent doses in combination with conventional therapy. In these patients the dose of PTH (1–34) varied between 20 mcg 4–6 times per week and one patient was only taking 10 mcg three times per week (9.1%). Additional details are provided in Supplementary Table 9. There were no significant differences in BMD values at the lumbar spine (L1–L4) between PTH (1–34) users and those solely on conventional therapy (*p* = 0.511). However, femoral neck (*p* = 0.050), total hip (*p* = 0.044), and 1/3 radius (*p* = 0.050) BMD values were lower in PTH (1–34) users compared to non-PTH users. Regarding serum calcium levels, Tables 5, 6 indicates no significant differences in serum corrected calcium or ionized calcium between PTH (1–34) users and non-PTH users.

**Table 5:** Comparison of BMD values between PTH (1–34) Users and Non-PTH users

Variable	Group	*N*	Mean ± SD	Range	*p*-value
L1-L4 BMD (gm/cm^2^)	PTH (1–34) users	10	1.168 ± 0.361	0.815–1.920	0.511
	Non-PTH users	75	1.218 ± 0.203	0.738–1.516	
Femoral neck BMD (gm/cm^2^)	PTH (1–34) users	10	0.876 ± 0.193	0.630–1.256	0.050
	Non-PTH users	78	1.002 ± 0.189	0.542–1.393	
Total hip BMD (gm/cm^2^)	PTH (1–34) users	10	0.930 ± 0.215	0.680–1.344	0.044
	Non-PTH users	77	1.070 ± 0.202	0.669–1.516	
1/3 radius BMD (gm/cm^2^)	PTH (1–34) users	9	0.757 ± 0.141	0.483–0.976	0.050
	Non-PTH users	71	0.851 ± 0.131	0.491–1.212

**Table 6:** Comparison of serum calcium (total, corrected, and ionized) levels between PTH (1–34) users and non-PTH users

Variable	Group	*N*	Mean ± SD	Range	*p*-value
Total calcium	PTH (1–34) users	11	2.164 ± 0.157	1.920–2.380	0.987
	Non-PTH users	90	2.162 ± 0.230	1.150–2.590	
Corrected calcium	PTH (1–34) users	11	2.103 ± 0.152	1.820–2.340	0.720
	Non-PTH users	90	2.078 ± 0.218	1.150–2.490	
Ionized calcium	PTH (1–34) users	11	1.142 ± 0.096	0.990–1.260	0.950
	Non-PTH users	80	1.140 ± 0.104	0.800–1.390	

## Discussion

The main finding of this study is that a systematic assessment of skeletal health in chronic HypoPT patients receiving conventional therapy revealed significant bone fragility in postmenopausal women compared to premenopausal women with chronic HypoPT. Our study revealed a high proportion of fractures and osteoporosis, as measured by BMD criteria, in postmenopausal women (35%) and men ≥ 50 (33.3%). We also observed a high prevalence of low BMD in postmenopausal women (25%). The prevalence of osteoporosis by fragility fractures or BMD is higher than reported in eucalcemic postmenopausal women (35% vs. 21.1%, respectively) [32]. This suggests that HypoPT may not provide skeletal protection in this patient population as may have been previously perceived. This baseline data from a prospective study of 101 patients from the CNHR provides a valuable comprehensive overview of the etiology, biochemical profile, and skeletal health status of the adult chronic HypoPT population in Canada. Overall, baseline demographic and clinical characteristics of the population, as well as the prevalence of postsurgical and non-surgical chronic HypoPT, were consistent with published data from studies of patient registries conducted in North America, Europe, and Russia [33–36].

Although most patients were receiving calcium and active vitamin D supplements for the treatment of chronic HypoPT, signs and symptoms of hypocalcemia were frequently reported. In line with previous studies, this suggests that the disease is not adequately controlled with conventional therapy in a significant proportion of patients, which can eventually lead to an increased burden of illness and increased risk of long-term complications [33, 37–40]. Novel therapies for chronic HypoPT are aimed at restoring normal PTH physiology, either by PTH replacement or using PTHR1 agonists. Recent phase 2 and phase 3 clinical trials evaluating palopegteriparatide (TransCon PTH) have reported that this molecule effectively maintained normocalcemia while providing independence from conventional therapy and improving health-related quality of life [24, 25]. The PTHR1 agonist (eneboparatide) has been shown to maintain serum calcium within the target range while normalizing urinary calcium excretion and producing a balanced resumption of bone turnover in a phase 2 trial [26].

The biochemical profile analysis by menopausal status revealed some differences between premenopausal and postmenopausal women, yet both profiles were overall comparable. The 25(OH)D levels were higher in postmenopausal than premenopausal women, suggesting better supplementation in the former, though both were maintained within the target range between 75 and 125 nmol/L. On the other hand, a significantly lower eGFR in postmenopausal women was observed compared to premenopausal women reflecting impairment of renal function with age. Notably, postmenopausal women also had hypercalciuria and nephrolithiasis, which suggests that this population could be at increased risk of renal complications. The serum ALP was significantly higher (*p* = 0.040) in postmenopausal women in comparison to premenopausal women consistent with the increased bone turnover expected postmenopause [41]. Our data also revealed statistically significantly lower TSH levels (*p* = 0.018) in the postmenopausal women compared to the premenopausal women within the normal reference range in our cohort. This may be a confounding factor to the lower BMD results observed in postmenopausal women.

Chronic HypoPT has been associated with BMD values that are above average compared to age- and sex-matched healthy subjects [42, 43]. Our study showed that nearly 12% of patients had a fragility fracture(s) and 83% of whom were postmenopausal women, consistent with recently published studies [14, 43, 44]. In the cross-sectional study of Mendonca et al., an increased frequency of morphometric vertebral fractures was shown in a small cohort of postmenopausal women with chronic HypoPT compared with age-matched healthy women [14]. More recently, a study by Cipriani et al. revealed a higher prevalence of vertebral fractures in postmenopausal women with chronic HypoPT compared with age-matched healthy postmenopausal women (16% vs. 7.5%) [43]. Of note, mean BMD values of postmenopausal women enrolled in our study are comparable to that of postmenopausal women reported by Cipriani et al. [43]. Reported fracture rates were even higher (up to 31%, mainly nonclinical morphometric) in a recent single-center study involving postsurgical chronic HypoPT, mostly women with a mean age of 64 years [44].

We previously assessed the risk of osteoporotic fracture in the CNHR population and reported that approximately 14% of chronic HypoPT patients were at high risk of major osteoporotic fracture [6]. In the current study with a systematic assessment of BMD and TBS, we identified 25% of postmenopausal women with chronic HypoPT as having low BMD and 31.3% have osteoporosis by BMD criteria, whereas BMD was normal in all of the premenopausal women. The prevalence of osteoporosis was higher in postmenopausal women with chronic HypoPT than in premenopausal women or men under age 50 years. Indeed, almost all of the patients with osteoporosis at the lumbar spine, femoral neck, or 1/3 radius were postmenopausal women. These findings underscore a high prevalence of osteoporosis and low BMD in postmenopausal women with chronic HypoPT, suggesting that skeletal health is impaired in this population.

The validity of TBS to estimate bone quality in chronic HypoPT has been evaluated in numerous studies, especially related to postmenopausal osteoporosis. Cipriani et al. reported that the mean TBS values in a chronic HypoPT population of diverse etiology remained within the normal range (1.44 ± 0.12), even when women were divided into postmenopausal and premenopausal groups [17]. In the study of Sakane et al., a population of postsurgical chronic HypoPT patients showed a normal mean TBS value (1.39 ± 0.14), although one-third of the results were below the normal range. Notably, abnormal TBS correlated with osteopenia, type 2 diabetes mellitus, low-impact fracture, and menopause [45]. Cipriani et al. reported TBS scores in the “degraded” range in postmenopausal chronic HypoPT and healthy women, suggesting predictability for vertebral fracture [43] In the study of Saha et al., TBS correlated with menopausal status and indicated degraded bone microarchitecture in 50% of the postmenopausal women [46]. In line with these data, postmenopausal women with chronic HypoPT in this study exhibited a significant reduction in mean TBS as compared to premenopausal women, further suggesting impaired bone strength.

Our study has limitations, mainly related to the observational design of a registry study as well as the lack of a control group. We are presenting the baseline data from this study. Although some data are missing for certain parameters, the CNHR is ongoing and will continue collecting patient data for future analyses.

Further research will determine the impact of long-term PTH, PTH analogues, and PTHR1 agonists on skeletal health in patients with chronic HypoPT especially in postmenopausal women and men over 50. Our study strongly suggests that a systematic timely evaluation of skeletal health by DXA assessment and spinal imaging would be useful to better assess fracture risk and bone strength in chronic HypoPT.

## Conclusions

The results of the baseline data from this prospective cohort study demonstrated significant bone fragility in postmenopausal women with chronic HypoPT of the CNHR, as shown by a higher-than-expected proportion of low BMD, osteoporosis, and/or fragility fractures. Similarly, a large percentage of men over the age of 50 years had osteoporosis by BMD criteria or prior fragility fracture. These findings suggest that, contrary to common belief, close follow-up of skeletal health is necessary in postmenopausal women with chronic HypoPT and men over the age of 50 years.

## Supplementary Information

Below is the link to the electronic supplementary material.

Supplementary file1 (DOCX 46 KB)

## Data Availability

The datasets generated during and/or analyzed during the current study are not publicly available but are available from the corresponding author on reasonable request.
