# Supplementary material for: Skeletal health status among patients with chronic hypoparathyroidism: results from the Canadian National Hypoparathyroidism Registry (CNHR)
Source: Osteoporos Int. 2025 Feb 16;36(4):673–84. doi: 10.1007/s00198-025-07410-7 (PMC12064612; doi:10.1007/s00198-025-07410-7)
Supplement: Supplementary file 1 — Supplementary file1 (DOCX 46 KB) [file 198_2025_7410_MOESM1_ESM.docx]

supplementary material

**Supplementary Table 1.** Serum Calcium (Total, Corrected, and Ionized) in Participants with and without Paresthesia

| Variable | Paresthesia | N | Mean ± SD | P-value |
| --- | --- | --- | --- | --- |
| Serum Total Calcium | No | 55 | 2.17 ± 0.21 | 0.643 |
|  | Yes | 45 | 2.15 ± 0.24 |  |
| Corrected Calcium | No | 55 | 2.09 ± 0.21 | 0.627 |
|  | Yes | 45 | 2.07 ± 0.22 |  |
| Ionized Calcium | No | 49 | 1.14 ± 0.09 | 0.592 |
|  | Yes | 41 | 1.13 ± 0.12 |  |

**Supplementary Table 2.** Serum Calcium (Total, Corrected, and Ionized) in Participants with and without Numbness

| Variable | Numbness | N | Mean ± SD | P-value |
| --- | --- | --- | --- | --- |
| Serum Total Calcium | No | 60 | 2.18 ± 0.22 | 0.560 |
|  | Yes | 40 | 2.15 ± 0.24 |  |
| Corrected Calcium | No | 60 | 2.09 ± 0.21 | 0.581 |
|  | Yes | 40 | 2.07 ± 0.22 |  |
| Ionized Calcium | No | 54 | 1.15 ± 0.10 | 0.499 |
|  | Yes | 36 | 1.13 ± 0.11 |  |

**Supplementary Table 3.** Comparison of Biochemical Markers Between Osteoporotic and Non-Osteoporotic T-scores among men over 50 years

| Variable | Osteoporotic  T-score | N | Mean ± SD | P-value |
| --- | --- | --- | --- | --- |
| Serum Calcium | No | 8 | 2.17 ± 0.16 | 0.501 |
|  | Yes | 1 | 2.05 |  |
| Corrected Calcium | No | 8 | 2.10 ± 0.13 | 0.718 |
|  | Yes | 1 | 2.15 |  |
| Ionized Calcium | No | 8 | 1.14 ± 0.10 | 0.688 |
|  | Yes | 1 | 1.09 |  |
| Phosphorus | No | 7 | 1.35 ± 0.20 | 0.081 |
|  | Yes | 1 | 1.79 |  |
| 25 hydroxyvitamin D | No | 8 | 92.83 ± 28.07 | 0.203 |
|  | Yes | 1 | 51.00 |  |
| Magnesium | No | 8 | 0.78 ± 0.08 | 0.161 |
|  | Yes | 1 | 0.92 |  |

**Supplementary Table 4.** Comparison of Biochemical Markers Between Osteoporotic and Non-Osteoporotic T-scores among postmenopausal women

| Variable | Osteoporotic  T-score | N | Mean ± SD | P-value |
| --- | --- | --- | --- | --- |
| Serum Total Calcium | No | 30 | 2.23 ± 0.17 | 0.311 |
|  | Yes | 10 | 2.29 ± 0.12 |  |
| Corrected Calcium | No | 30 | 2.15 ± 0.14 | 0.306 |
|  | Yes | 10 | 2.21 ± 0.13 |  |
| Ionized Calcium | No | 26 | 1.18 ± 0.12 | 0.659 |
|  | Yes | 10 | 1.16 ± 0.06 |  |
| Phosphorus | No | 30 | 1.30 ± 0.24 | 0.411 |
|  | Yes | 9 | 1.37 ± 0.22 |  |
| 25 hydroxyvitamin D | No | 29 | 105.70 ± 28.45 | 0.309 |
|  | Yes | 10 | 115.90 ± 21.05 |  |
| Magnesium | No | 30 | 0.80 ± 0.07 | 0.260 |
|  | Yes | 10 | 0.83 ± 0.20 |  |

**Supplementary Table 5.** Biochemical variables and bone markers among female and male HypopT patients

|  | **Women** | | **Men** | | **P-value** |
| --- | --- | --- | --- | --- | --- |
|  | Mean ± SD | Range | Mean ± SD | Range |  |
| **Total calcium (mmol/L)** | 2.18 ± 0.23  (n=83) | 1.15 – 2.59 | 2.10 ± 0.17  (n=18) | 1.78 – 2.48 | 0.215 |
| **Corrected calcium (mmol/L)** | 2.10 ± 0.22  (n=83) | 1.15 – 2.49 | 2.01 ± 0.17  (n=18) | 1.66 – 2.32 | 0.140 |
| **Ionized calcium (mmol/L)** | 1.15 ± 0.10  (n=75) | 0.80 – 1.39 | 1.11 ± 0.10  (n=16) | 0.94 – 1.32 | 0.240 |
| **Creatinine (µmol/L)** | 77.86 ± 44.90  (n=83) | 39.00 – 430.00 | 126.11 ± 152.08  (n=18) | 72.00 – 733.00 | **0.015** |
| **Calcium phosphate product** | 2.85 ± 0.57  (n=75) | 1.40 – 4.30 | 2.90 ± 0.41  (n=15) | 2.40 – 3.85 | 0.779 |
| **Magnesium (mmol/L)** | 0.81 ± 0.08  (n=80) | 0.53 – 1.04 | 0.78 ± 0.08  (n=17) | 0.62 – 0.92 | 0.292 |
| **Phosphate (mmol/L)** | 1.36 ± 0.24  (n=77) | 0.74 – 2.00 | 1.45 ± 0.24  (n=15) | 1.12 – 1.81 | 0.182 |
| **eGFR (mL/min)** | 82.66 ± 23.55  (n=83) | 8.00 – 120.00 | 82.50 ± 26.16  (n=18) | 6.00 – 117.00 | 0.980 |
| **ALP (U/L)** | 72.87 ± 25.83  (n=76) | 10.00 – 151.00 | 72.71 ± 25.54  (n=17) | 43.00 – 120.00 | 0.981 |
| **PTH (pmol/L)** | 1.74 ± 1.48  (n=80) | 0.30 – 11.20 | 1.96 ± 1.84  (n=17) | 0.35 – 8.10 | 0.599 |
| **TSH (mL/L)** | 2.70 ± 3.59  (n=81) | 0.01 – 18.80 | 2.14 ± 1.28  (n=17) | 0.57 – 5.02 | 0.526 |
| **25(OH)D (nmol/L)** | 99.44 ± 25.98  (n=78) | 44.00 – 164.00 | 86.79 ± 30.96  (n=17) | 37.00 – 128.20 | 0.082 |
| **1,25(OH)D (pmol/L)** | 101.37 ± 38.32  (n=43) | 23.00 – 216.00 | 61.82 ± 31.15  (n=11) | 20.00 – 119.00 | 0.003 |
| **CTX (ng/L)** | 306.74 ± 412.49  (n=58) | 65.00 – 3051.00 | 333.91 ± 234.98  (n=11) | 143.00 – 991.00 | 0.833 |
| **P1NP (µg/L)** | 43.26 ± 36.34  (n=57) | 10.00 – 215.00 | 35.08 ± 8.40  (n=12) | 22.00 – 54.00 | 0.444 |
| **24 h Urine calcium (mmol/day)** | 5.28 ± 4.90  (n=66) | 0.26 – 30.40 | 5.24 ± 3.66  (n=15) | 1.03 – 13.72 | 0.973 |

1,25(OH)D, 1,25-dihydroxyvitamin D; 25(OH)D, 25-hydroxyvitamin D; ALP, alkaline phosphatase; CTX, collagen type-I telopeptide; eGFR, estimated glomerular filtration rate; P1NP, procollagen 1 N-terminal propeptide; PTH, parathyroid hormone; TSH, thyroid stimulating hormone. Bold indicates statistically significant p-values.

**Supplementary Table 6.** Biochemical variables and bone markers among non-surgical and postsurgical HypoPT patients

|  | **Non-surgical** | | **Postsurgical** | | **P-value** |
| --- | --- | --- | --- | --- | --- |
|  | Mean ± SD | Range | Mean ± SD | Range |  |
| **Total calcium (mmol/L)** | 2.11 ± 0.26  (n=26) | 1.28 – 2.42 | 2.18 ± 0.21  (n=75) | 1.15 – 2.59 | 0.138 |
| **Corrected calcium (mmol/L)** | 2.01 ± 0.26  (n=26) | 1.28 – 2.34 | 2.11 ± 0.19  (n=75) | 1.15 – 2.49 | **0.040** |
| **Ionized calcium (mmol/L)** | 1.11 ± 0.12  (n=24) | 0.80 – 1.30 | 1.15 ± 0.10  (n=67) | 0.85 – 1.39 | 0.115 |
| **Calcium phosphate product** | 2.74 ± 0.60  (n=23) | 1.48 – 4.24 | 2.90 ± 0.52  (n=67) | 1.40 – 4.30 | 0.215 |
| **Magnesium (mmol/L)** | 0.79 ± 0.09  (n=25) | 0.53 – 0.93 | 0.81 ± 0.08  (n=72) | 0.55 – 1.04 | 0.281 |
| **Phosphate (mmol/L)** | 1.36 ± 0.26  (n=24) | 0.77 – 1.81 | 1.38 ± 0.24  (n=68) | 0.74 – 2.00 | 0.763 |
| **eGFR (mL/min)** | 80.15 ± 27.45  (n=26) | 8.00 – 120.00 | 83.49 ± 22.68  (n=75) | 6.00 – 120.00 | 0.542 |
| **ALP (U/L)** | 70.25 ± 30.83  (n=24) | 31.00 – 151.00 | 73.74 ± 23.76  (n=69) | 10.00 – 132.00 | 0.569 |
| **PTH (pmol/L)** | 1.90 ± 2.22  (n=24) | 0.30 – 11.20 | 1.74 ± 1.26  (n=73) | 0.30 – 8.10 | 0.678 |
| **TSH (mL/L)** | 2.15 ± 1.58  (n=24) | 0.60 – 8.42 | 2.75 ± 3.70  (n=74) | 0.01 – 18.80 | 0.444 |
| **25(OH)D (nmol/L)** | 92.34 ± 29.38  (n=24) | 37.00 – 164.00 | 98.81 ± 26.44  (n=71) | 44.00 – 162.00 | 0.316 |
| **1,25(OH)D (pmol/L)** | 98.00 ± 33.48  (n=17) | 49.00 – 171.00 | 91.16 ± 43.01  (n=37) | 20.00 – 216.00 | 0.565 |
| **CTX (ng/L)** | 477.27 ± 732.88  (n=15) | 144.00 –3051.00 | 264.91 ± 204.12  (n=54) | 65.00 – 1015.00 | 0.061 |
| **P1NP (µg/L)** | 36.29 ± 16.57  (n=16) | 17.00 – 82.00 | 43.51 ± 36.86  (n=53) | 10.00 – 215.00 | 0.452 |
| **24 h Urine calcium (mmol/day)** | 4.31 ± 3.15  (n=20) | 0.47 – 12.27 | 5.59 ± 5.05  (n=61) | 0.26 – 30.40 | 0.293 |

1,25(OH)D, 1,25-dihydroxyvitamin D; 25(OH)D, 25-hydroxyvitamin D; ALP, alkaline phosphatase; CTX, collagen type-I telopeptide; eGFR, estimated glomerular filtration rate; P1NP, procollagen 1 N-terminal propeptide; PTH, parathyroid hormone; TSH, thyroid stimulating hormone. Bold indicates statistically significant p-values.

**Supplementary Table 7.** BMD and TBS by sex

|  | **Women** | | **Men** | | | **P-value** |
| --- | --- | --- | --- | --- | --- | --- |
|  | **Mean ± SD** | **Range** | | **Mean ± SD** | **Range** |  |
| **Total hip BMD** (gm/cm^2^) | 1.019 ± 0.194  (n=71) | 0.669 – 1.476 | | 1.207 ± 0.196  (n=16) | 0.731 – 1.516 | **0.001** |
| **L1-L4 BMD** (gm/cm^2^) | 1.193 ± 0.212  (n=69) | 0.738 – 1.753 | | 1.292 ± 0.269  (n=16) | 0.996 – 1.962 | 0.116 |
| **Femoral neck BMD** (gm/cm^2^) | 0.957 ± 0.185  (n=72) | 0.542 – 1.393 | | 1.125 ± 0.167  (n=16) | 0.817 – 1.363 | **0.001** |
| **1/3 radius BMD** (gm/cm^2^) | 0.814 ± 0.116  (n=67) | 0.483 – 0.990 | | 0.972 ± 0.149  (n=13) | 0.736 – 1.212 | **<0.0001** |
| **TBS score^a^** | 1.342 ± 0.146  (n=58) | 0.968 – 1.622 | | 1.313 ± 0.153  (n=11) | 0.968 – 1.482 | 0.556 |

BMD, bone mineral density; L, lumbar spine; TBS, trabecular bone score; SD, standard deviation. ^a^Degraded TBS is ≤1.23, partially degraded is >1.23 but ≤1.31, and normal TBS is >1.31. Bold indicates statistically significant p-values.

**Supplementary Table 8.** BMD and TBS by etiology

|  | **Non-surgical** | | **Postsurgical** | | | **P-value** | |  |
| --- | --- | --- | --- | --- | --- | --- | --- | --- |
|  | **Mean ± SD** | **Range** | | **Mean ± SD** | **Range** | |  | |
| **Total hip BMD** (gm/cm^2^) | 1.107 ± 0.189  (n=22) | 0.755–1.387 | | 1.035 ± 0.211  (n=65) | 0.669 – 1.516 | | 0.158 | |
| **L1-L4 BMD** (gm/cm^2^) | 1.285 ± 0.184  (n=22) | 0.815–1.538 | | 1.186 ± 0.234  (n=63) | 0.738 – 1.962 | | 0.076 | |
| **Femoral neck BMD** (gm/cm^2^) | 1.047 ± 0.189  (n=22) | 0.664–1.336 | | 0.967 ± 0.190  (n=66) | 0.542 – 1.393 | | 0.093 | |
| **1/3 radius BMD** (gm/cm^2^) | 0.859 ± 0.144  (n=21) | 0.483–1.212 | | 0.833 ± 0.131  (n=59) | 0.491 – 1.393 | | 0.456 | |
| **TBS score^a^** | 1.409 ± 0.095  (n=17) | 1.226–1.562 | | 1.314 ± 0.153  (n=52) | 0.968 – 1.622 | | **0.018** | |

BMD, bone mineral density; L, lumbar spine; TBS, trabecular bone score; SD, standard deviation. ^a^Degraded TBS is ≤1.23, partially degraded is >1.23 but ≤1.31, and normal TBS is >1.31. Bold indicates statistically significant p-values.

**Supplementary Table 9.** Baseline characteristics of PTH users (n=11)

| Variable | N | % |
| --- | --- | --- |
| Gender | | |
| *Females* | 10 | 90.91 |
| *Males* | 1 | 9.09 |
| Menopause Status  (Female N=10) | | |
| *Premenopausal* | 2 | 20.00 |
| *Postmenopausal* | 8 | 80.00 |
| Etiology | | |
| *Post-surgical* | 7 | 63.64 |
| *Non-surgical* | 4 | 36.36 |
| Number of patients on PTH1-84 | 0 | 0.00 |
| Number of patients on PTH1-34 | 11/101 | 10.89 |
| Dosage regimen |  |  |
| *20 mcg daily* | 4 | 36.36 |
| *20 mcg 4-7x/week* | 1 | 9.09 |
| *20 mcg 5-7x/week* | 1 | 9.09 |
| *10 mcg 3x/week* | 1 | 9.09 |
| *Missing* | 4 | 36.36 |
|  | **N** | **Mean ±SD** |
| Age (yrs) | 11 | 64.18 ± 16.19 |
| Duration of chronic HypoPT (yrs) | 11 | 17.09 ±11.29 |
